# Dehydroepiandrosterone Effect on *Toxoplasma gondii*: Molecular Mechanisms Associated to Parasite Death

**DOI:** 10.3390/microorganisms9030513

**Published:** 2021-03-02

**Authors:** Saé Muñiz-Hernández, Angélica Luna-Nophal, Carmen T. Gómez-De León, Lenin Domínguez-Ramírez, Olga A. Patrón-Soberano, Karen E. Nava-Castro, Pedro Ostoa-Saloma, Jorge Morales-Montor

**Affiliations:** 1 Laboratorio de Oncología Experimental, Subdirección de Investigación Básica, Instituto Nacional de Cancerología, Secretaria de Salud, Ciudad de México 14080, Mexico; sayide@hotmail.com (S.M.-H.); angitaluna87@gmail.com (A.L.-N.); 2 Departamento de Inmunología, Instituto de Investigaciones Biomédicas, Universidad Nacional Autónoma de México, AP 70228, Ciudad de México 04510, Mexico; carmen_tgl@live.com.mx (C.T.G.-D.L.); postoa@unam.mx (P.O.-S.); 3 Departamento de Ciencias Químico-Biológicas, Escuela de Ciencias, Universidad de las Américas Puebla, Santa Catarina Mártir, Cholula, Puebla 72810, Mexico; julio.dominguez@udlap.mx; 4 División de Biología Molecular, Instituto Potosino de Investigación Científica y Tecnológica, Camino a la Presa San José 2055, Col. Lomas 4a. Sección, San Luis Potosí 78216, Mexico; araceli.patron@ipicyt.edu.mx; 5 Laboratorio de Genotoxicología y Mutagénesis Ambientales, Departamento de Ciencias Ambientales, Centro de Ciencias de la Atmósfera, Universidad Nacional Autónoma de México, Ciudad de México 04510, Mexico; karlenc@atmosfera.unam.mx

**Keywords:** Toxoplasmosis, *Toxoplasma gondii*, tachyzoite, antiparasitic effect, dehydroepiandrosterone, DHEA, proteomic profile, protection

## Abstract

Toxoplasmosis is a zoonotic disease caused by the apicomplexa protozoan parasite *Toxoplasma gondii*. This disease is a health burden, mainly in pregnant women and immunocompromised individuals. Dehydroepiandrosterone (DHEA) has proved to be an important molecule that could drive resistance against a variety of infections, including intracellular parasites such as *Plasmodium falciparum* and *Trypanozoma cruzi*, among others. However, to date, the role of DHEA on *T. gondii* has not been explored. Here, we demonstrated for the first time the toxoplasmicidal effect of DHEA on extracellular tachyzoites. Ultrastructural analysis of treated parasites showed that DHEA alters the cytoskeleton structures, leading to the loss of the organelle structure and organization as well as the loss of the cellular shape. In vitro treatment with DHEA reduces the viability of extracellular tachyzoites and the passive invasion process. Two-dimensional (2D) SDS-PAGE analysis revealed that in the presence of the hormone, a progesterone receptor membrane component (PGRMC) with a cytochrome b5 family heme/steroid binding domain-containing protein was expressed, while the expression of proteins that are essential for motility and virulence was highly reduced. Finally, in vivo DHEA treatment induced a reduction of parasitic load in male, but not in female mice.

## 1. Introduction

Toxoplasmosis is a zoonosis caused by the Apicomplexa protozoan parasite, *Toxoplasma gondii*, which is able to infect all warm-blooded animals [1,2]. This is a worldwide disease with a prevalence average of 40% [3]. Particularly, in Mexico, the sero-prevalence goes from 40 to 70% depending on the region of the country [4,5]. *T. gondii* infection can induce abortion, encephalitis, and in extreme cases, death. Indeed, it is considered a major opportunistic pathogen in patients with AIDS [6,7].

Human toxoplasmosis presents two phases: the acute and the chronic one. In the acute phase, the parasite disseminates in the tachyzoite stage, which is the highly invasive and motile asexual form. In this stage, the parasite is able to cross any biological barrier, included the placenta or the blood–brain barrier [8,9,10,11]. In most immunocompetent hosts, tachyzoites will eventually differentiate into bradyzoites, the low replication form, and will begin the tissue cyst formation [12]; this event defines the chronic infection, since tissue cysts can stay forever in the host without provoking any apparent pathology [13].

The tachyzoite stage has a characteristic half-moon shape and an approximate size of 5 to 10 μm [14], as all members of the Apicomplexa family. The parasite’s motility depends on actomyosin machinery that underlies the plasma membrane called the glideosome [15]. *Toxoplasma* has three specialized secretory organelles with particular proteins, which are secreted in a controlled and specific manner as parasite biological needs: the micronemes (MIC protein), rhoptries (ROP proteins), and dense granules (GRA proteins) [16].

The *Toxoplasma* tachyzoite can carry out two types of invasion, active or passive. Active invasion is the process by which all nucleated and non-phagocytic host cells are infected. Active invasion has been widely studied and is dependent on the well-orchestred, lytic cycle [13,17,18,19,20]. The less studied, passive invasion is the process by which all phagocytic cells are invaded. First, the parasite adheres to the plasma membrane of a phagocytic activated cell, which is surrounded by the plasmatic membrane elongations, and it is internalized toward the cytoplasm in a phagocytic vacuole [13]. Once inside, the parasite evades the immune response, transforming the phagocytic vacuole into a parasitophorous vacuole (PV) via the phosphorylation of the host Immune-Related GTPases (IRGs) by a complex that involves ROP and GRA proteins. This prevents IRG’s oligomerization and their recruitment to the PVM, hampering the vacuole lysis, and as a consequence, the clearance of the parasites by the macrophages [13,21,22,23].

Conventional therapy against toxoplasmosis consists of a mixture of sulfadiazine–pyrimethamine (S-P) that was established in the 1950s. Since then, minor advances have been made in the treatment of this zoonosis [24,25]. Although sulfadiazine–pyrimethamine are synergic, it is known that it does present severe side effects. Since pyrimethamine is a folic acid antagonist, it has been associated with bone marrow toxicity, while sulfadiazine causes hypersensitivity and allergic reactions in up to 20% of the population [26,27]. Furthermore, this conventional treatment has a limited effectiveness, mainly on the chronic stage of the disease, and there is not an available vaccine for human or veterinary use.

Dehydroepiandrosterone (DHEA) is a steroid hormone that is produced from cholesterol, in the adrenal glands, gonads, and brain, and it is synthesized from pregnenolone by the action of the 17, 20-desmolase enzyme [28]. It is the most abundant hormone circulating in mammals and can also be a precursor of sexual steroids [29]. The sulfated form of DHEA is majorly found in blood circulation and the free DHEA form (the active form) is only 3–5% of the total concentration. Although DHEA is a hormone produced by many organisms, it has proven to be an excellent as an antiparasitic agent. In vitro, low concentrations of DHEA inhibit the proliferation, adhesion, and motility of *Entamoeba histolytica* trophozoites, while high concentrations induce the lysis of the parasite [30]. DHEA reduces 75% of the reproduction of *Taenia crassiceps* cysticercus in vitro; and in the murine model infected with metacestodes of *Taenia*, the parasite load was 50% reduced when mice were previously treated with the hormone [31]. Particularly, in a previous study, DHEA was administered pre and post-infection with *T. gondii* to immunosuppressed mice; this reduced mortality by 65% and 50%, respectively. Furthermore, both treatments reduced the number of brain cysts when compared to the control [32]. 

In the present study, we assessed the effect of DHEA, alone or in combination with the conventional treatment S-P, on *Toxoplasma gondii* extracellular tachyzoites and in a mice model of acute toxoplasmosis. Our results suggest that DHEA may be recognized by a progesterone receptor membrane component (PGRMC) with a cytochrome b5 family heme/steroid binding domain-containing protein, inducing a reduction of passive invasion by the modulation of the expression of proteins that are essential during the invasive process, as well as some virulence factors. Moreover, we observed that DHEA have an important role decreasing the parasitic load in mice. 

## 2. Materials and Methods

### 2.1. Drugs, Reagents, and Solutions

DHEA (cat.809640, Sigma Chemical Co. Steinheim, Germany) was dissolved in anhydrous ethanol (cat. 459836, Sigma chemical Co. Steinheim, Germany). The sulfadiazine–pyrimethamine was obtained in its commercial formulation (Bactropin ^®^, trimethoprim-sulfamethoxazole 160/800 mg, Laboratorios Quimica Son’s, S.A. de C.V., Mex.). All salts were purchased from J.T. Baker (Phillipsburg, NJ, USA) NaCl, cat.3524-01; K_2_PO_4_ cat.7758-11-4; Na_2_HPO_4_ cat. 3828-01 and KCl cat. 3040-01. Others reagents were purchased from Sigma (Sigma Chemical Co. Steinheim, Germany): Mineral oil cat.M5904, glucose cat.604-68-2, lipopolysaccharide cat. L3129, glutaraldehyde cat. G7651, Osmium tetroxide (OsO4) cat. 658685, uranyl acetate cat. 6159-44-0, urea cat. U5128, Tris-HCL cat. T3253, Dodecyl-Sulfate Sodium (SDS) cat. L3771, glycerol cat. G5516, and silver nitrate cat. 2091-39.

### 2.2. Animals

Male Balb-C/AnN mice, 6–8 weeks old, were used for parasite infection and were maintained in a pathogen-free environment with regulated conditions of temperature, humidity, and filtered air at National Cancer Institute Facilities. Animals were fed with autoclaved food and water ad libitum and maintained according to the Mexican Federal Regulations for Animal Production, Care, and Experimentation (NOM-062-ZOO-1999, Ministry of Agriculture; Mexico City, Mexico). All efforts to minimize animal suffering and to reduce the number of animals used were made.

### 2.3. Maintenance and Purification of T. gondii Tachyzoites

Tachyzoites of the RH strain were maintained by intraperitoneal (ip) passages in six-week-old Balb/cAnN male mice. After euthanazia, parasites were recovered from peritoneal exudates, washed with 1X PBS (138 mM NaCl, 1.1 mM K_2_PO_4_, 0.1 mM Na_2_HPO_4_ and 2.7 mM KCl, pH 7.2), and purified by filtration through 5 μm pore polycarbonate membranes (cat. TMTP-02500, Merck Millipore Co. Cork, Ireland).

### 2.4. Murine Macrophage Culture

Sterile mineral oil (1 mL) was inoculated in the peritoneum of male BalbC/AnN mice. After five days, the mice were euthanized, and intraperitoneal macrophages were recovered using 1% glucose-PBS solution. Macrophages were centrifuged, and the pellet was resuspended in Dulbecco’s Modified Eagle Medium (cat. D5696, DMEM, Gibco, Thermo Fisher, NY, USA), supplemented with 8% of fetal bovine serum (cat. 35-015-CV, FBS, Corning, NY, USA) and 1% of penicillin–streptomycin (PES 100 u/mL, cat. 15070-063 Thermo Fisher, NY, USA). Macrophages were seeded over sterile coverslips in a ratio of 250 × 10^3^/cm^2^, and they were maintained in a 5% CO_2_ atmosphere at 37 °C.

### 2.5. Macrophage Activation

Purified macrophages of mice were washed with fresh DMEM; they were seeded and maintained by 24 h in incubation before the activation. Lipopolysaccharides (LPS, Sigma Chemical Co., Steinheim, Germany) were added at 30 ng/mL during 1 h in order to activate the macrophages. Then, they were invaded with tachyzoites as described in the “Invasion assays” section.

### 2.6. Viability of Extracellular Tachyzoites

Purified parasites (6 × 10^6^ cells) were exposed to increasing concentrations of DHEA (1, 10, 20, 50, 80, 100, and 200 μM), of sulfadiazine–pyrimethamine (80, 200, 400, 600, and 800 μM), and to the combined treatment DHEA/S-P (80/80, 200/200, 10/600 and 10/800 μM). All drugs were diluted in PBS 1X to the final concentrations tested. The exposition was held for 30 min or 120 min at room temperature (RT) with gentle agitation. Additionally, we used a control of ethanol (Sigma), as the primary diluent of DHEA, at a final concentration of 2 µM. The ratio between live and death tachyzoites was measured by the trypan blue exclusion technique (cat. 15250–061, Gibco, Thermo Fisher, NY, USA). Three hundred parasites were counted under an optical microscope (AxioObserve A.1 Microscope, Carl Zeiss Mexico). This assay was performed in triplicate in at least three independent assays.

### 2.7. Invasion Assay

Activated or non-activated macrophages were exposed to 6 × 10^6^ tachyzoites during 120 min, after which samples were washed, fixed with formaldehyde (10%) (cat. 2106-01, J.T. Baker, NJ, USA) for 30 min, and stained with hematoxylin–eosin (Hematox, cat.CATHE-M, Biocare Medical Co, CA, USA; Eosin, cat.36720, Golden Bell Ind., Mex). Stained parasites were analyzed in an optical microscope. The invasion process was evaluated counting 300 total cells; we considered as an invaded cell every one that presented at least one parasitophorous vacuole into the cell cytoplasm. Quantitative analysis was performed in an AxioObserve A.1 microscope (Carl Zeiss, Mexico, Mexico). This assay was done by triplicated in at least three independent assays.

### 2.8. Analysis of Tachyzoite Morphology in Response to DHEA 

Extracellular tachyzoites treated with DHEA, S-P, or DHEA/S-P at several concentrations, and at 30 or 120 min of incubation time, were processed for Transmition Electron Microscopy (TEM). Briefly, tachyzoites were resuspended in 2.5% glutaraldehyde (Sigma) in 1× PBS in gentle agitation for 1 h, washed with 1× PBS, fixed with 1% OsO4 1 h (Sigma), and contrasted with 1% aqueous uranyl acetate during 120 min (Sigma). Samples were dehydrated at increasing concentrations of ethanol (50–100%) and then were embedded in LR White resin (cat. 14380, London Resin- England, Electron Microscopy Sciences, PA, USA) and polymerized at 4 °C during 36 h under a UV lamp. The samples were cut with an ultramicrotome. Serial cuts were performed at around 10 μm of thickness and mounted in a sample holder; the ultrastructural analysis was performed in a Transmission Electron Microscope JEM200CX 200KV (JEOL Co., Tokyo, Japan), and image analysis was performed using the Digital Micrograph program (TM 3.7.0 for GSM 1.2 by the Gatan Software Team).

### 2.9. 2D SDS-PAGE

Whole extracts of intact or treated parasites with DHEA (10 μM), S-P (800 μM), and DHEA/S-P (10/800 μM) exposed for 30 min was obtained by lysis in 2D sample buffer; extracts were centrifuged at 10,000 rpm, soluble fractions were quantified in a NanodropTM 2000 (Thermo Scientific) at 280 nm. Then, 100 μg of whole extracts, contained in 125 μL of rehydratation buffer, were loaded on ImmobilineTM DryStrip pH 3–10, 7 cm strips (cat. GE17600114, GE Healthcare, IL, USA). After 16 h of passive rehydratation, an isoelectric focus was performed in a Protean IEF Cell (Bio-Rad Laboratories, Firmware Version: 1.80) by the supplier specifications. Strips were equilibrated in an equilibrium buffer (6 M urea, 0.375 M Tris-HCl pH 8.8, 2% SDS, 20% glycerol) with 0.5% dithiothreitol (DTT, cat.1610611, BioRad Lab., CA, USA) for 10 min, and then with equilibrium buffer with 2.5% iodoacetamide (IAA, cat.1632109, BioRad Lab., Hercules, CA, USA) for another 10 min; then, strips were loaded in polyacrylamide precast gels (Mini-PROTEAN^®^TGXTM Precast Gels 4–20%, cat.4561094, Bio-Rad Lad., Hercules, CA, USA) and electrophoresis was performed at 100 V; then, gels were stained with silver nitrate (Sigma) and scanned in a HP Scanjet G4050 scanner.

### 2.10. In Silico Analysis of 2D SDS-PAGE

Three independent experiments were performed with different treatments. Then, the generation of a master gel of each experimental condition was performed in order to identify the differential spots and proteins among them. Once the spots were identified, they were compared both by isoelectric point and by molecular weight with the PDQuestTM software (Bio-Rad Laboratories, Inc). Then, the deduction of the possible identity of each protein that exhibited differential expression between treatments was carried out using the Expert Protein Analysis System (ExPASy) bioinformatics platform (https://web.expasy.org/tagident/, accessed on 9 January 2021) [33], considering a range of ±0.1 values of each isoelectric point. Likewise, the deduction of the probable protein function was identified by the UniProtKB platform (https://www.uniprot.org/statistics/Swiss-Prot, accessed on 9 January 2021).

### 2.11. Modelling, Docking, and Molecular Dynamics of the Progesterone Receptor Membrane Component (PGRMC) 

Initial model generation was accomplished by using the cloned sequence for *Toxoplasma gondii* PGRMC and submitting it to Rosetta Homology modeling [34]. Resulting models clustered close together for the selection of the best model. However, since the template is a PGRMC1 protein in complex with a heme group (PDB ID 4X8Y) [35], these models were refined using UCSF Chimera-Modeller plugin [36,37]. Then, their quality was evaluated using Molprobity [38]. The highest quality model was selected to perform ligand docking. Blind docking was performed using Vina 1.1.2 on the LNS supercomputer. All ligands were obtained from the ZINC database and converted to PDBQT format using the GUI provided by Autodock Tools. The receptor was kept rigid during docking. Docking employed a grid of dimensions 40 × 40 × 40 with a 1 Å grid size. Exhaustiveness was always set to 1000. Analysis of the docking results was performed in UCSF Chimera. The results presented in Table 2 (PBS 1x) are the best candidates selected from the consensus score with the three best results.

### 2.12. In Vivo Treatment of Acute Toxoplasmosis in the Mice Model

Male and Female Balb-C/AnN mice, between 6 and 8 weeks of age were infected with 10 × 10^3^ tachyzoites of *Toxoplasma*. The treatment (DHEA, S-P, or DHEA/S-P) was administered at unique concentration for each drug (200 µg/100 µL) diluted in corn oil, via oral using a cannula. The doses were administered daily starting day 2 after infection with the parasite, during 6 days. After that time, the mice were humanely euthanized; intraperitoneal exudate was obtained with 10 mL PBS1X washes. Then, it was centrifugated, and the pellet was resuspended in PBS1X, free parasites were counted with trypan blue exclusion technique, in a Neubauer chamber on an AxioObserve A.1 microscope (Carl Zeiss, Mexico). Each experimental group of both male and female mice consisted of 8 individuals. All animals were maintained as was previously described.

### 2.13. Statistical Analysis

Statistical analysis was performed with a variance analysis (ANOVA) of two ways that allowed determining simultaneously the effect of two variables (treatment and exposition time) with the Tukey comparison proof. We used the program GraphPad Prism 6, analysis was considered significantly different when *p* < 0.05. Analysis of the in vivo experiments considered an experimental design with 3 independent variables: sex (two levels: male or female), DHEA exposure (Control or DHEA), and infection (I). Data from 2 independent experiments were analyzed with the Prism 6^®^ software (GraphPad Software Inc.) and charted as mean ± standard deviation. Data distribution normality was determined with a Shapiro–Wilk test. Thereafter, a one-way ANOVA (α = 0.05) was performed followed by a Tukey post-hoc test (*p* < 0.05).

## 3. Results

### 3.1. The Treatment with DHEA Decreases the Viability of Toxoplasma Gondii Extracellular Tachyzoites 

**Figure 1:**
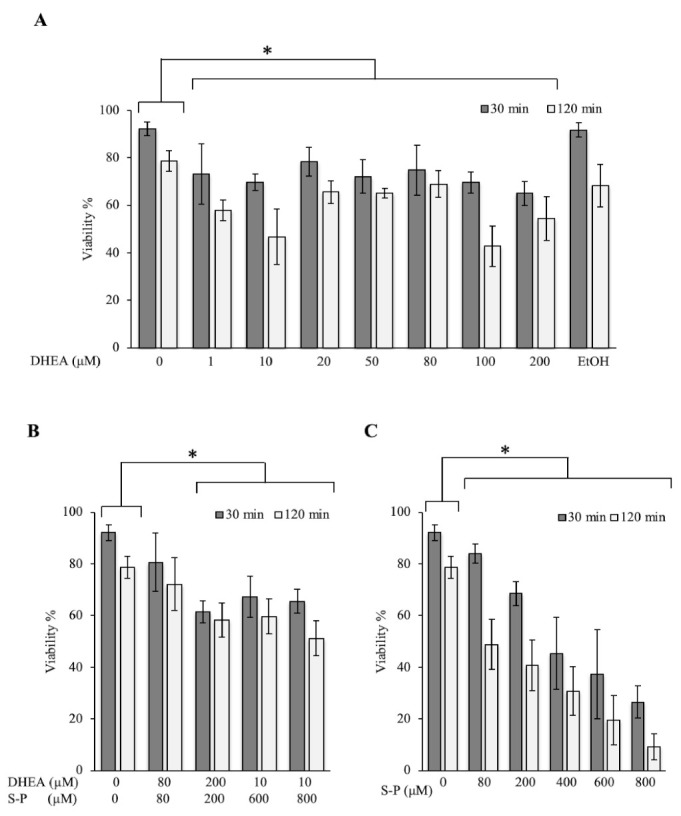
Effect of dehydroepiandrosterone (DHEA) on *T. gondii* extracellular tachyzoites viability. (**A**) DHEA (**B**) DHEA/sulfadiazine-pyrimethamine (S-P) and (**C**) S-P treatment. The *x*-axis shows the final concentration of each drug; the *y*-axis shows the percentage of viability. The “0” value corresponds to tachyzoites without treatment (only PBS); EtOH corresponds to the DHEA solution vehicle (ethanol at 2 μM final concentration). (*) Statistical significance compared to the control according to exposure time. *p* < 0.0001.

In order to know the effect of DHEA on the viability of Toxoplasma, we exposed extracellular tachyzoites to increasing DHEA concentrations during 30 or 120 min. In the viability assay, all concentrations used induced a considerable decrease in the parasite viability at both times tested. At 30 min, the decrease observed was between 15 and 30%, while at 120 min, the decrease was around 13–50% compared to control conditions (Figure 1A). The maximum effect was observed for the 10 and 100 μM, which are concentrations that reduced viability by about half (Figure 1A). These results suggest that the viability of extracellular tachyzoites of Toxoplasma is compromised when they are exposed to increasing concentrations of DHEA.

### 3.2. The Combined Treatment (DHEA/S-P) Did Not Reach the Effect on Tachyzoites Viability Compared to Conventional Treatment

In order to assess the possibility of using DHEA as an auxiliary compound in the treatment against Toxoplasma infection, we tested the effect of the conventional treatment S-P in combination with the hormone. A viability diminution of approximately 30% with 200 μM concentration at both times tested was observed (Figure 1B). With the conventional treatment (S-P), the effect observed at 200 μM resulted in approximately a 25% viability decreased at 30 min; however, at 120 min, it reaches 50% of viability inhibition (Figure 1C). The effect of DHEA (100 μM-120 min) was similar to S-P alone (200 μM-120 min) (Figure 1A vs. Figure 1C). Parasites were also treated with a constant concentration of 10 μM of DHEA combined with 600 or 800 μM of S-P. Under these combinations, parasites viability showed a reduction of approximately 30% (Figure 1B). This effect was similar to 10 μM DHEA alone treatment. Unfortunately, its combination not achieve the effect of S-P used as individual treatment (Figure 1B vs. Figure 1C). In accordance with these data, in combined treatment, DHEA appears to inhibit S-P activity. The viability inhibition in parasites exposed to S-P exhibited an almost linear behavior for all concentrations used and for both exposition times, the maximum concentration induced near 90% of parasite mortality (Figure 1C, 800 μM).

### 3.3. Treatment with DHEA Induces Changes in the Proteomic Profile

**Figure 2:**
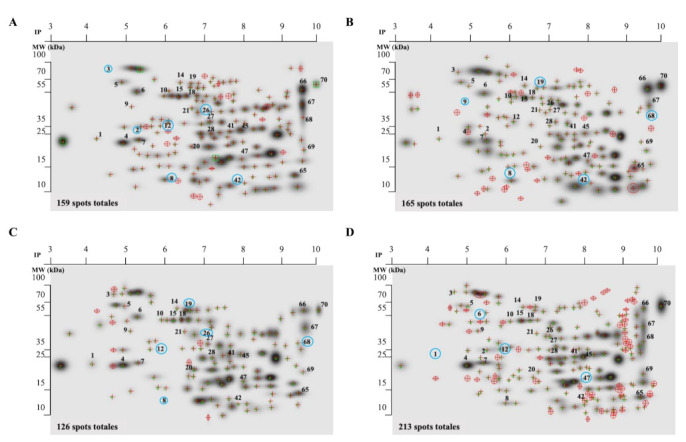
Proteomic profile of the whole extract of extracellular tachyzoites at 30 min of treatment. (**A**) Control without treatment; (**B**) DHEA 10 μM; (**C**) S-P 800 μM, and (**D**) DHEA 10 μM/S-P 800 μM. The red crosses represent the spots located in each treatment, numbers in the spots represent the proteins analyzed (listed in Table 1), and the blue circles represent the missing proteins in each condition.

We determine the protein pattern expression of extracellular tachyzoites in the presence of DHEA, S-P, and DHEA/S-P compared to non-treated parasites. According to the protein profile of total extracts, untreated tachyzoites exhibited 159 spots, while the tachyzoites treated with DHEA (10 μM), S-P (800 μM), and DHEA/S-P (10/800 μM) exhibited 165, 126, and 213 spots, respectively (Figure 2A–D). We quantify the total number of spots that match in each condition with the control spots, and we found 105, 99, and 113 spots in DHEA (10 μM), S-P (800 μM), and DHEA/S-P (10/800 μM), respectively.

**Figure 3:**
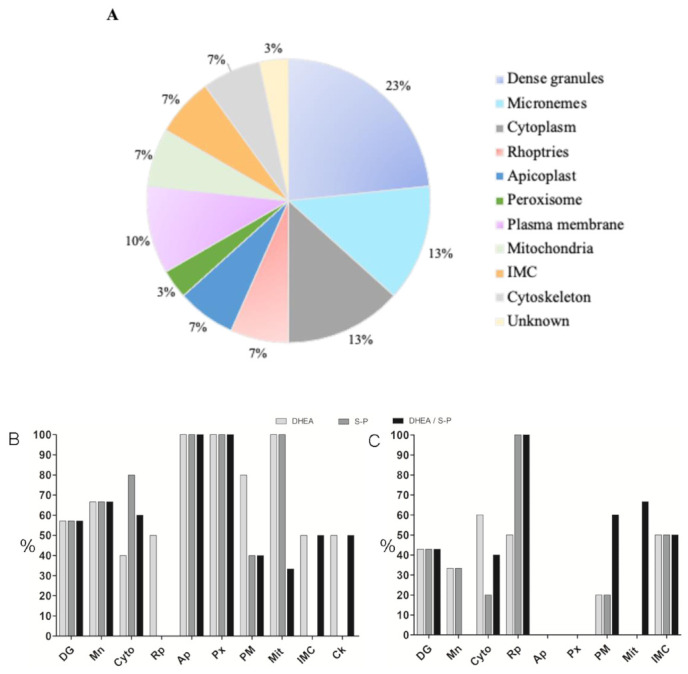
Classification of proteins that exhibited differential expression. (**A**) Proteins that change their expression after 30 min of treatment with DHEA 10 μM, S-P 800 μM, or DHEA/S-P 10/800 μM, grouped by their probable location. (**B**) Percentage of proteins that increase their expression with respect to the control without treatment (*y*-axis); in the *x*-axis, the proteins identified were arranged by their probable location; (**C**) Percentage of proteins that increase their expression with respect to the control without treatment (*y*-axis); in the *x*-axis, the proteins identified were arranged by their probable location: DG, dense granules; MIC, micronemes; ROP, rhoptries; API, apicoplast; PER, peroxisome; CK, cytoskeleton; CYT, cytoplasm; MIT, mitochondria; MEM, plasma membrane; IMC, inner membrane complex; and UNK, unknown localization.

The protein profiles were analyzed using the PDQuest software (Bio-Rad). We selected the proteins that showed greater changes in their expression among the treatments and with respect to the control. Thirty proteins were identified through their molecular weight and isoelectric point (Table 1), and they were classified according to their probable location (Figure 3A). Most proteins that changed their expression are dense granules proteins, which are followed by proteins from cytoplasm and micronemes (Table 1).

Then, we graphed the proteins that decreased, maintained, or increased their expression with respect to the control (Figure 3B). It was possible to observed that DHEA treatment alone leads to a slight increase in the expression of proteins from micronemes, cytoplasm, and rhoptries compared to other treatments (Figure 3B). Meanwhile, S-P treatment provokes a protein expression reduction in most of them, with the exception of dense granules (GRA1), micronemes (MIC2), and cytoskeleton (profilin) (Figure 3B). Combined treatment with DHEA/S-P induces a lower expression of proteins from dense granules, micronemes, cytoplasm, apicoplast, and peroxisome, and a higher expression of GRA3, GRA7 (GD), and rhomboid-like protease (ROM3) (plasma membrane, MEM) (Figure 3).

### 3.4. Interaction of DHEA with the Progesterone Receptor Membrane Component (PGRMC) a Cytochrome b5 Family Heme/Steroid Binding Domain-Containing Protein

Interestingly, the spot number 2, which exhibited an experimental molecular weight of 27 kDa and isoelectric point of 5.1, and it is expressed only in the protein profile of tachyzoites treated with DHEA alone or DHEA/S-P combined, was identified as a progesterone receptor membrane component (PGRMC): a cytochrome b5 family heme/steroid binding domain-containing protein with a theoretical molecular weight of 26.25 kDa and isoelectric point of 5.18. The primary sequence of the protein was aligned in the NCBI web site with the BLASTprogram (https://blast.ncbi.nlm.nih.gov/Blast.cgi?PROGRAM=blastp&PAGE_TYPE=BlastSearch&LINK_LOC=blasthome, access on 9 January 2021), and the protein was aligned with other steroid binding proteins from other eukaryotes (Figure S1A–C). Then, we aligned only the domain section (aminoacids from 129 to 176), and the result was similar.

**Figure 4:**
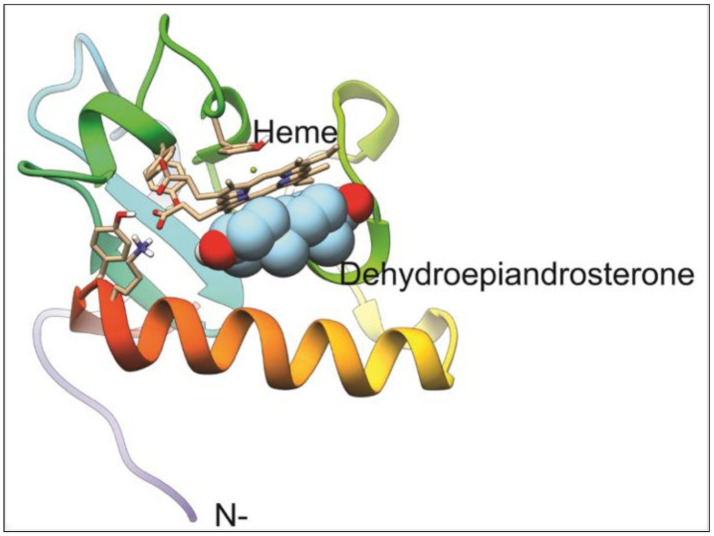
Model for *T. gondii* progesterone receptor membrane component (PGRMC) homolog and its docking to DHEA. The model for PGRMC contains a binding pocket for a heme group that functions as the binding site for DHEA. TYR158 binds the heme group on one face, while the other binds DHEA, blocking any interaction at that site.

The *T. gondii* model generated is the best that can be obtained given that the only available template has a 36.9% homology. This corresponds to the 111 residues located at the carboxyl-terminus of the full 243 residue protein. Most of the residues known to interact with the heme group in the PGRMC1 structure are identical in our predicted structure for *T. gondii* PGRMC. The resulting model has a heme group partially buried and contributing significantly to the binding of all of the ligands tested (Figure 4). In every case, the three best results for each ligand were in contact with the heme group on the surface of the protein. Notably, progesterone is the most tightly bound ligand followed by DHEA, testosterone, and 4–5 alpha dihydrotestosterone (Table 2). Given that residue TYR158 (numbering based on the whole sequence cloned) provides the fifth coordination to the heme-iron, forcing ligand interaction to occur on the unoccupied side of the heme group. Pyrimethamine and sulfadiazine were also found to bind the heme group but with significantly lower affinity (Table 2, asterisks). Given that these affinities are about 1.5 kcal/mol lower, it is likely that they are non-specific as well, as is the case for fatty acids included in the docking. It is unknown if *T. gondii* PGRMC is able to dimerize as its template (Homo sapiens PGRMC1), but the interactions with the ligands tested in the present work would block or compete a similar interaction.

### 3.5. The Treatment with DHEA Reduces the Passive Invasion Process

**Figure 5:**
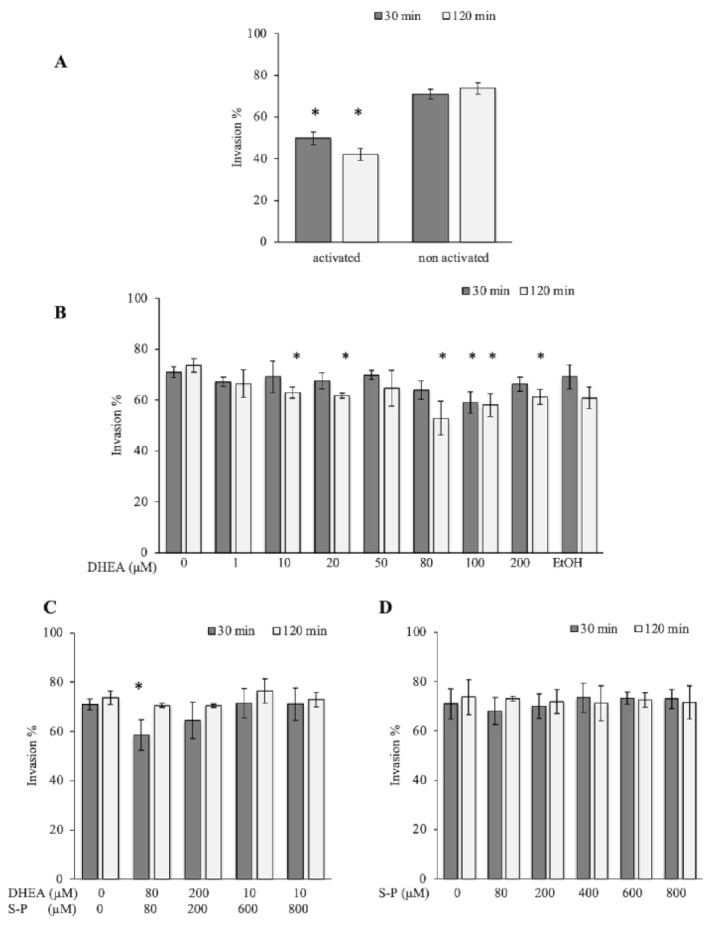
Effect of DHEA in the passive invasion process. (**A**) Activated vs. Unactivated lipopolysaccharides (LPS) macrophages (**B**) DHEA treatment (**C**) DHEA/S-P, and (**D**) S-P treatment; on the *x*-axis, the final concentration of each drug is plotted; while on the *y*-axis, the percentage of macrophages that contained at least one parasitophorous vacuole (PV) in the cellular cytoplasm is plotted. EtOH corresponds to DHEA solution vehicle (ethanol 2 μM final concentration). (*) Statistical significance compared to the control according to exposure time. *p* < 0.05 compared to the control according to exposure time. *p* < 0.05.

*T. gondii* has the capability to invade all nucleated cells, including phagocytic cells such as macrophages. The parasite uses the active machinery of the phagocytic cells to invade; this kind of invasion is called passive. GRA7 expression was reduced when parasites were treated with DHEA, while S-P treatment exhibited a similar expression to the control and in an unexpected way, the combined treatment with DHEA/S-P induced a slight expression increase (Table 1). Based on the hypothesis that GRA7 has a role during passive invasion, we assess if DHEA treatment has an effect on the passive invasion process. Fresh macrophages were confronted with pre-treated extracellular tachyzoites for 30 min or 120 min (Figure 5). First, we compared the percentage of phagocytized untreated tachyzoites by LPS-activated macrophages, compared to the percentage of phagocytized untreated tachyzoites by non-activated macrophages. Activated macrophages phagocytized between 20 and 40% more than the non-activated macrophages (Figure 5A). For this reason, we use activated macrophages for the consecutive assays. Passive invasion was reduced between 15 and 30% when activated macrophages were exposed to DHEA pre-treated tachyzoites for 120 min (Figure 5B). The maximal invasion inhibition was observed to 80 µM/120 min (*p* = 0.007, IC 95%). The combined (DHEA/S-P) and the conventional (S-P) treatments on extracellular tachyzoites have no effect on the passive invasion, independently of the concentration and time (Figure 5C,D respectively). However, we can observe a slight decrease around 12% at 80/80 µM DHEA/S-P at 30 min only, which could be an effect of DHEA.

### 3.6. Morphological Changes in Extracellular Tachyzoites Induced by DHEA 

**Figure 6:**
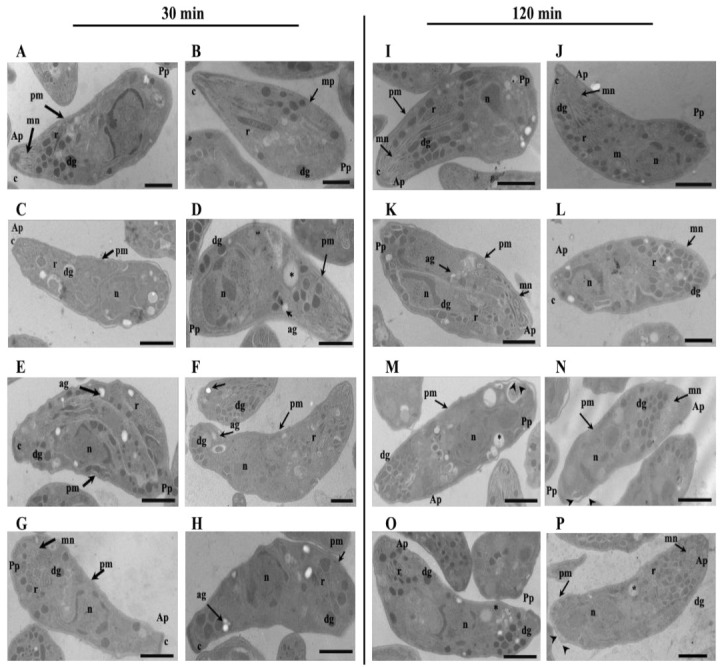
Effect of DHEA in the ultrastructure of Toxoplasma gondii extracellular tachyzoites. Morphology of extracellular tachyzoites drug exposed by (**A**–**H**) 30 min and (**I**–**P**) 120 min. (**A**,**I**) untreated tachyzoites; (**B**,**J**) tachyzoites exposure to ethanol. Extracellular tachyzoites exposure to DHEA (**C**,**K**) 10 μM, (**D**,**L**), 100 μM, DHEA/S-P (**E**,**M**) 10/600 μM, (**F**,**N**) 10/800 μM; or (**G**,**O**) 200 μM, ang (**H**–**P**) 800 μM. Ap, apical pole; Pp, posterior pole, c, conoid; mn, micronemes, r, rhoptries; dg, dense granules; pm, plasma membrane; ag, amylopectin granules; n, nucleus; asterisks, empty cytoplasmic vacuoles; arrow heads, waviness and separation of parasite plasma membrane. Bars = 1 µm.

We analyzed if the change in the protein expression and decrease in the proliferation process could be related to morphological changes induced by the DHEA treatment on extracellular tachyzoites. The ultrastructure images of extracellular parasites treated as in the viability assay, for all concentrations of each treatment, DHEA or S-P alone and DHEA/S-P combined, were obtained by TEM (Figure 6A–P). Images of extracellular parasites treated for 30 min (Figure 6A–H) and 120 min (Figure 6I–P) are presented in Figure 6. Untreated and vehicle control (ethanol) tachyzoites are shown in Figure 6A,B,I,J. The DHEA treatment at 10 μM for 30 min preserves all the typical structures such as micronemes (mn), rhoptries (r), dense granules (dg), nucleus (n), mitochondria (m), and some areas of the plasmatic membrane (pm) look wavy (Figure 6 C). At 100 μM DHEA at 30 min, the parasites seems to lose their typical half-moon shape, and some of them present dense granules in the posterior pole (Figure 6D). Importantly, the effect of DHEA on the tachyzoites structure is related to the concentration and time used; a longer time of DHEA exposition induced greater changes in the extracellular tachyzoites morphology. Parasite exposure to 10 and 100 μM of DHEA at two hours showed an amoeboid shape with a total loss of the intracellular organization and the apical polarity. The dense granules lose their circle shape, and the presence of some amylopectin granules were observed, too (Figure 6K,L, respectively). In the combined treatment, DHEA/S-P at 10/600 μM and 10/800 μM, the effect was observed starting at 30 min (Figure 6E,F) of exposure, and it was consistent after 120 min (Figure 6 M,N). At 10/600 μM DHEA/S-P, the parasites no longer have their typical shape and present an amoeboid and elongated shape; they also seem to lose the intracellular organization and present an important number of amylopectin granules (Figure 6E,M). At higher concentrations of DHEA/S-P 10/800 μM, some parasites preserved their typical shape and lose the organization of the organelles; the rhoptries are now at the posterior pole, the dense granules lose their circled shape, and they present an important number of empty cytoplasmic vacuoles (Figure 6F,N). The conventional treatment induced important changes in the extracellular tachyzoites, independently of concentration or time exposure (Figure 6G,H,O,P). At 200 and 800 μM of S-P for 30 min and 120 min, the tachyzoites lose their typical shape, and it was possible to find tachyzoites with amoeboid, elongated, or amorphous shape, with no apical polarity, and no organization of the organelles (Figure 6G,H,O,P).

### 3.7. DHEA Treatment Decreased the Parasite Load in BalbC/ANn Male Mice

**Figure 7:**
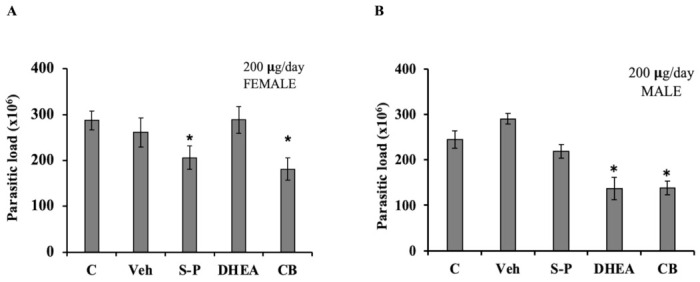
Effect of DHEA in acute infection of *Toxoplasma gondii*. Mice previously infected with tachyzoites of *T. gondii* were administered with 200 µg/day of DHEA or S/P or 200µg/day of DHEA/S-P. (**A**) Female mice and (**B**) Male mice; the *y*-axis shows the parasite load obtained in the intraperitoneal exudate. (*) showed the statistical significance compared to untreated group. C, control (untreated mice), Veh, corn oil was used as vehicle; CB, combined treatment. *p* < 0.05.

Finally, we analyzed if DHEA treatment could be used in acute *T. gondii* in vivo infection. We used both female and male mice in order to take into account the role of endogenous hormone level. DHEA or S-P or DHEA/S-P were administered at 200 µg/mouse, daily over 5 days, in mice previously infected with Toxoplasma; after treatment, mice were euthanized, and the parasitic load was quantified. We obtained peritoneal exhudate, and free tachyzoites were purified and counted (Figure 7). According with our data, DHEA did not have an effect on the parasite load in infected female mice (Figure 7A). However, female mice treated with the conventional treatment exhibited a low parasitic load (*p* = 0.033), in the same way as the combined treatment (*p* = 0.007, CI 95%). In male mice, DHEA treatment induced an important reduction in the parasite load (*p* = 0.012). This reduction was also observed in the combined treatment (*p* = 003, CI 95%) (Figure 7B). In male mice, the DHEA administration induced a reduction of 50% in the parisite load, and this effect was also observed in DHEA/S-P combined treatment adminsitration (Figure 7B). Moreover, although the parasite load observed in S-P was slightly lower than that of the control or vehicle group, it did not reach statistical significance (Figure 7B).

## 4. Discussion

DHEA induces a decrease in the viability of extracellular tachyzoites at 100 μM at both times tested (30 and 120 min), even if the effect was lower than that observed with standard therapy (S-P); results are similar to those obtained when trypomastigotes of *T. cruzi*, another intracellular parasite, were treated with 128 μM of DHEA combined with melatonin for 24 h [39]. Different to the standard treatment, whose response was directly proportional to the concentration, the effect obtained with DHEA exposure was independent on the concentration. This suggests that both drugs have different targets inside of the tachyzoite. In order to evaluate an accumulative effect of the DHEA with S-P, we tested the combined treatment, resulting in a similar effect as that obtained when DHEA alone was administered. It is important to mention that previous studies have reported that DHEA’s antiparasitic effect depends on the administration scheme, experimental conditions, and parasite lineage [30,31].

After bidimensional electrophoresis protein separation, the following in silico analysis showed a cytochrome b5 family heme/steroid binding domain-containing protein, only under DHEA exposition (alone or S-P combined). Given its homology to PGRMC 1 and 2, which are proteins known for their role as progesterone receptors, as well as interactions with the family of cytochromes P450 monooxygenase systems [40], it is not surprising to find it associated to a drug metabolism and response function in *T. gondii*. Interaction between this PGRMC homolog and DHEA could potentially block normal activating interactions with CYPs, thus preventing the removal of the steroid. Moreover, the experiment in cells, as well as the molecular docking, provides evidence for DHEA to have a different target and effect than S-P. Furthermore, in all sequenced parasites genomes, there are not specific proteins to bind steroid hormones, but there is a very conserved PGRMC. Thus, we extend the notion that parasites use a protein to bind steroids, with different affinities.

During the active invasion, *Toxoplasma* is the effector cell and the recognition of an unknown component in the plasma membrane of the host cell is required. This event is determined by the GPI-anchored proteins from *Toxoplasma*, of which the SAG1 protein is the most abundant in the plasma membrane of the tachyzoites [13]. The expression of SAG1 was reduced in parasites that were treated with any drug (DHEA or S-P or DHEA/S-P). It is well known that SAG1 is not the only protein that acts in the process of attachment, since Sag1 mutants are still able to invade [13]. The motility is essential for the invasion and it depends of the glideosome complex; in this respect, several proteins that participate in the formation of the glideosome and in its correct function reduced its expression when parasites were treated with DHEA and DHEA/S-P, such as GAP40, profilin, and ROM4. The GAP40 protein acts as an anchor for the rest of the glideosome complex [41]. The role of profilin is to sequester the G actin in order to enhance the polymerization, and it has been demonstrated to be essential for the gliding motility and cell cycle in *Toxoplasma* [42]. ROM4 is a rhomboid protease that is necessary for the cleavage of the complex MIC2/AMA1 that is formed for the establishment of the moving junction (MJ) during the invasion process. The correct cleavage of the complex is required for correct reorientation of the parasite and gliding motility [43]. The reduction of the expression of these proteins leads us to suppose that DHEA would modify the parasites’ ability to invade their host cells. However, in the present work, we do not evaluate the active invasion process; complementary experimental assays are needed to support this notion.

Once we determined that extracellular tachyzoites viability was affected by DHEA treatment, we assessed if the hormone has an effect on the passive invasion process. One theory about the molecular mechanisms that *T. gondii* uses in order to avoid the macrophages lysis is that GRA7 interacts with the ROP18 kinase in a complex that targets the host IRGs, mediating macrophage survival and acute virulence [44].

The analysis in primary cultures of macrophages revealed that high concentrations of DHEA affect the tachyzoites’ ability to establish in the cytoplasm of the phagocytic cell. In comparison with DHEA treatment, neither the conventional (S-P) nor the combined treatment (DHEA/S-P) presented significant differences, even when the combined treatment reduced the extracellular viability. It has been proposed that tachyzoites can transform the phagocytic vacuole into a parasitophorous vacuole by two different processes: the first includes the formation of the moving junction (MJ) at the same time that the parasite is phagocytized, whilst the second implies that once the parasite has been phagocytized, it is able to invade the phagolysosomal vacuole [13,44]. Both theories imply the fusion of the tachyzoite plasma membrane with the macrophage plasma membrane or with the phagolysosome membrane. This mechanism involves the secretion of proteins from secretory organelles, such as MIC2 and RON4 [18]. Interestingly, the MIC2 protein exhibited a higher expression when extracellular tachyzoites were exposed to DHEA; its expression was lower in the S-P exposure and undetectable in the combined treatment. Indeed, the expression of RON4 was higher in the DHEA treatments compared to the other treatments. It has been previously reported that extracellular tachyzoites that were exposed to progesterone inhibited the secretion of MIC2, but they did not affect its expression [45]. This inhibition of the secretion leads to the inhibition of gliding motility. In this work, we did not collected the secretion products, but they have to be collected in future assays in order to determine if these proteins are secreted or not. Nevertheless, the normal level of MIC2 expression observed in the protein extracts from tachyzoites treated with DHEA vs. treated with S-P in the 2D assays could be due to a failure on its secretion.

Proliferation assays revealed that although the parasites were established inside of the macrophages, the evasion of the lysis was inhibited by DHEA treatment, which could prevent the block of the phosphorylation of the host Immune-Related GTPases (IRGs) by ROP18 and GRA7, which are proteins from the parasite, that decrease its ability to escape lysosomal degradation. Concomittant to this, the expression of GRA7 was reduced when parasites were treated with DHEA, while S-P treatment exhibited a similar expression to the control. In an unexpected way, the combined treatment with DHEA/S-P increased the expression of the protein. GRA7 interacts with the ROP18 kinase in a complex that targets the host IRGs, mediating macrophage survival and acute virulence. For instance, the ∆GRA7 strain reduces the virulence by half, and the parasites can not evade the lysosomal degradation [46]. The protein expression changes, which again suggests that there are specific targets into the parasite for DHEA and S-P.

The effect of DHEA in the structure of the extracellular tachyzoites resulted in the alteration of the cytoplasmic organization of the organelles as well as the plasmatic membrane, secretory organelles and cytoskeleton structures. Tachyzoites that were treated with S-P and DHEA/S-P showed increased structural alterations, except for the swollen shape. The morphological changes induced in the tachyzoites by DHEA in our study are concordant with the morphological changes observed in the wall of *Toxoplasma* cysts [45]. Interestingly, GRA3 expression was enhanced when parasites were exposed to DHEA and DHEA/S-P. Recently, it was reported that GRA3 may have a role in the stabilization of the subpellicular cytoskeleton network, as ∆GRA3 strain tachyzoites-purified cytoskeletons lose the organization of this structure [47], which could be a possible explanation of why more parasites treated with DHEA/S-P preserve their characteristic form compared to tachyzoites treated with DHEA alone. 

The loss of the structure and location of secretory organelles when parasites were treated with DHEA could be in concordance with the reduction in the invasion and the ability to escape the macrophage lysis, because both mechanisms depend on the secreted proteins from micronemes, rhoptries, and dense granules. This effect is also related to the modifications in the expression of these proteins, as was previously discussed. 

Another two proteins with differential expression regulation that are worth mentioning are the diacylglycerol kinase catalytic domain-containing protein and enolase 2. The former is a protein that is essential for the correct secretion of micronemes [48]. This protein increases its expression in all treatments, incluiding DHEA. As we did not collect secretory products of the parasite, more experiments should be achieved in order to determine the effect of the hormone in the function of this protein.

Enolase 2, besides being specific to the tachyzoite stage, acts as a transcription factor during intracellular proliferation [49,50]. This protein maintains its expression similar to the control, when parasites were exposed to DHEA, while its expression was reduced with the S-P and DHEA/S-P treatment. Such expression could be associated to a major proliferation percentage observed in the intracellular tachyzoites pre-treated with DHEA.

It is worth noting that even though there is not evidence of sexual dimorphism in *Toxoplasma* infections, we found that the DHEA concentration used here in acute infections in immunocompetent mice exhibited an important parasiticidal effect solely in male mice. The DHEA effect was maintained even when it was combined with S-P. In this regard, we assume that DHEA exogenous administration in both males and females favors the synthesis of sex hormones testosterone and estrogen, respectively. Previously, in vitro assays showed that pre-treatment of tachyzoites of *T. gondii* with progesterone inhibited their capability to migrate to neighboring cells in contrast to tachyzoites pre-treated with estrogen, which had increased motility [45]. In a previous study, it was suggested that estradiol promotes both the pathogenicity and infection in Balb-c mice of both sexes infected with tachyzoites of *T. gondii* [51]; however, we do not observe this possible estrogen effect in female mice treated with DHEA, since the parasite load was similar with respect to control females. Indeed, our data with male mice are in agreement with that of a study from two decades ago where they reported a decrease in the *Toxoplasma* chronic infection of immunosuppressed mice who were administered with DHEA [32], although that work used an avirulent *Toxoplasma* strain. Taking together the previous results and those presented here, we could hypothesize that high levels of testosterone promote the activation of the immune system, leading to an inhibition in intracellular replication, consequently reducing the parasite loads. Finally, DHEA can be proposed as a new treatment by itself or in a combined scheme with conventional treatments; however, more experiments should be achieved in order to investigate its role as an antiparasitic drug in *Toxoplasma gondii* infections and the possible role of the immune system in the DHEA response in both acute and chronic infection.

## 5. Conclusions

The DHEA antiparasitic effect could be due to its interaction with the cytochrome b5 family heme/steroid binding domain-containing protein. DHEA treatment modifies the expression of proteins that are essential for the motility and virulence of RH strain tachyzoites, and it is likely to block the removal of DHEA by CYPs. This leads to an alteration of the ultrastructure of the parasites, the loss of the structure and organelles organization as well as of the cell shape. These alterations induce a reduction of the viability in vitro. Finally, the administration of DHEA in infected mice reduces the parasite load, solely in male mice (Table 3). Table 3 shows all results obtained to visualize and compared better the effect of DHEA, S-P, and DHEA-S-p on *Toxoplasma gondii.*

## Figures and Tables

**Table 1:** Differential expression of prroteins in whole extracts of extracellular *T. gondii* tachyzoites.

Spot	Protein Name	Uniprot KB ID	Theoretical Mr/IP	Experimental Mr/IP	Possible Location	Percentage of Expression			
						Ctrl	DHEA	S-P	DHEA/S-P
1	Dense granule protein (GRA1)	P13403	17.85/4.13	23/4.1	DG	100	103.43	116.11	_
2	Cytochrome b5 family heme/steroid binding domain-containing protein	A0A139XPA0	26.25/5.18	27/5.1	Unk	_	100.00	_	101.72
3	Microneme protein (MIC2)	O00816	82.61/4.45	84/4.5	Mn	100	100.00	89.70	-
4	Dense granule protein (GRA7)	O00933	23.23/4.95	22.9/4.9	DG	100	96.20	100.58	108.56
5	Microneme protein (MIC4)	Q9XZH7	63.02/4.84	63/4.66	Mn	100	102.02	100.16	86.91
6	Micronemal protein (MIC1)	O00834	46.97/5.20	52.7/5.15	Mn	100	111.72	80.88	-
7	Dense granule protein (GRA6)	Q27003	24.02/5.47	23.5/5.35	DG	100	111.37	83.77	100.28
8	Dense granule protein (GRA5)	Q07828	12.97/5.81	12.5/5.98	DG	100	111.56	-	87.52
9	Rhoptry protein (ROP1)	A0A125YP48	47.99/4.9	45/4.99	Rh	100	-	95.47	84.69
10	Enolase 2	Q9BPL7	48.29/5.67	53/5.71	Cp	100	97.69	77.87	82.51
12	Inosine-5′-monophosphate dehydrogenase	Q4VRV8	40.36/6.08	38.5/6.01	Cp	100	97.69	_	_
14	Rhoptry neck protein (RON4)	B6KJ32	65.34/6.49	65.8/6.41	Rh	100	103.18	93.99	87.09
15	Anamorsin homolog	B9Q0C2	47.56/6.49	53/6.38	Cp	100	80.32	67.19	73.99
18	Elongation factor Tu	Q9TMM9	44.31/6.52	52/6.5	Api	100	67.93	79.73	58.40
19	Peroxisomal catalase	Q9XZD5	57.27/6.71	63/6.69	Pxs	100	_	_	80.09
20	Profilin	A0A086PNN0	20.78/6.51	21/6.7	Ck	100	73.04	105.30	70.61
21	Gliding associated protein (GAP40)	E0AE39	43.08/6.61	44/6.6	IMC	100	96.97	97.93	82.98
26	Acid phosphatase GAP50	A0A086PXK7	46.60/6.95	46/6.97	IMC	100	100.00	_	74.67
27	Dense granule protein (GRA4)	Q27002	34.08/7.19	36.5/7.12	DG	100	67.36	67.59	62.95
28	Major surface antigen p30 (SAG1)	P13664	29.80/6.84	26/7.1	PM	100	72.51	68.20	63.38
41	Rhomboid-like protease (ROM1)	Q695U0	32.83/7.69	30/7.68	Mn	100	76.65	100.82	90.53
42	Actin depolymerizing factor (ADF)	A0A086PI60	12.94/7.92	12.5/7.8	Ck	100	_	100.00	93.97
45	Rhomboid-like protease (ROM3)	Q6IUY1	29.34/8.19	30/7.95	PM	100	79.51	76.99	104.23
47	Dense granule protein (GRA2)	P13404	17.46/8.21	17/8.0	DG	100	95.42	94.06	_
65	50S ribosomal protein L14	Q9XQQ6	14.08/9.77	13.9/9.4	Api	100	102.35	90.08	91.17
66	Rhomboid-like protease (ROM4)	Q695T8	69.66/9.24	65.5/9.51	PM	100	93.25	78.85	90.41
67	Cytochrome b	O20672	41.59/9.25	46.5/9.51	Mit	100	78.58	86.36	88.36
68	Phosphatidylserine decarboxylase proenzyme ^1^	Q1PCQ8	39.44/9.61	39/9.61	Mit	100	_	_	97.36
69	Dense granule protein (GRA3)	B6KEU8	24.24/9.46	20/9.65	DG	100	103.72	100.15	110.65
70	Diacylglycerol kinase catalytic domain-containing protein	A0A139XS45	69.58/10.02	63/9.97	Cp	100	145.63	97.38	136.91

^1^ Proteins were identified by molecular weight and isoelectric point using the program TagIdent in ExPASy web (https://web.expasy.org/tagident/, accessed on 9 January 2021). Expression level was normalized with control condition. All treatments were compared against the control (10 μM DHEA; 800 μM S-P; 10/800 μM DHEA/S-P). Mr; molecular weight, IP; isoelectric point; DG, dense granules; Unk, unknown; Mn, micronemes; Rh, rhoptries; Cp, cytoplasm; Api, apicoplast; Pxs, peroxisome; Ck, cytoskeleton; IMC, inner membrane complex; PM, plasma membrane; Mit, mitochondria.

**Table 2:** Ligands that presented best affinities to *Toxoplasma gondii* PGRMC.

Predicted Ligand	Theoretical Affinity(kcal/mol)
**4–5 alpha-Dihydrotestosterone**	**−7.4**
Aldosterone	−7.1
Beta-estradiol	−6.7
Cholesterol	−6.6
Corticosterone	−6.8
Cortisol	−6.5
Decanoate	−4.4
**DHEA**	**−7.4**
Dodecanoate	−4.6
Estriol	−7.2
Linoleate	−5.5
Myristate	−5
Octanoate	−4.1
Oleate	−5.4
Palmitate	−4.6
**Progesterone**	**−7.6**
Pyrimethamine	−5.9 *
Stearic	−5.1
Sulfadiazine	−5.5 *
**Testosterone**	**−7.4**

**Bold**, better affinities; (*), affinities of compounds of conventional treatment of Toxoplasma.

**Table 3:** Summary of all changes induced by DHEA, S-P, and DHEA/S-P treatments in *Toxoplasma gondii* in vitro and in vivo.

Experimental Assays	DHEA	S-P	DHEA/S-P
In vitro *viability Inhibition (%)*			
	15–30%, 30 min	25–72%, 30 min	25–40% 30 and 120 min
	13–50%, 120 min	>50%, 120 min	
**Proteomic profile**			
*Total number of spots*	165	126	213
*Number of spots that changed*	65	27	100
**Protein expression (N = 30)**			
*Overexpressed proteins*	9	5	4
*Subexpressed proteins*	12	16	18
*Absent proteins*	4	7	5
*Unchanged proteins*	3	5	3
**Particular protein expressed**	Cytochrome b5 family heme/steroid binding domain-containing protein		Cytochrome b5 family heme/steroid binding domain-containing protein
**BLAST analysis**	PGRMC homologue		PGRMC homologue
***In vitro* Passive invasion**			
*Inhibition (%)*	<6%, 30 min	No inhibition	≈12%
	15–30%, 120 min		≈30%
**Morphological Changes**	Lost typical half-moon shape, intracellular organization, and the apical polarity.	Tachyzoites exhibited an ameboid, elongated, or amorphous shape. Lost apical polarity and organelle organization.	Lost typical shape, intracellular organelle organization and polarity. Presence of amylopectin granules and/or empty cytoplasmic vacuole.
**In Vivo treatment** *Percent of reduction in peritoneal parasite load*			
Female mice	0%	≈28%	~37%
Male mice	45%	0%	44%

## Data Availability

The authors declare that all data supporting the findings of this study are available within the article and its Supplementary Information files.

## Supplementary Materials

The following are available online at https://www.mdpi.com/2076-2607/9/3/513/s1, Figure S1: Alignment of the cytochrome b5 family heme/steroid binding domain-containing protein.
