# Supplementary material for: Dehydroepiandrosterone Effect on Toxoplasma gondii: Molecular Mechanisms Associated to Parasite Death
Source: Microorganisms. 2021 Mar 2;9(3):513. doi: 10.3390/microorganisms9030513 (PMC8000356; doi:10.3390/microorganisms9030513)
Supplement: Supplementary file 1 [file microorganisms-09-00513-s001.pdf]

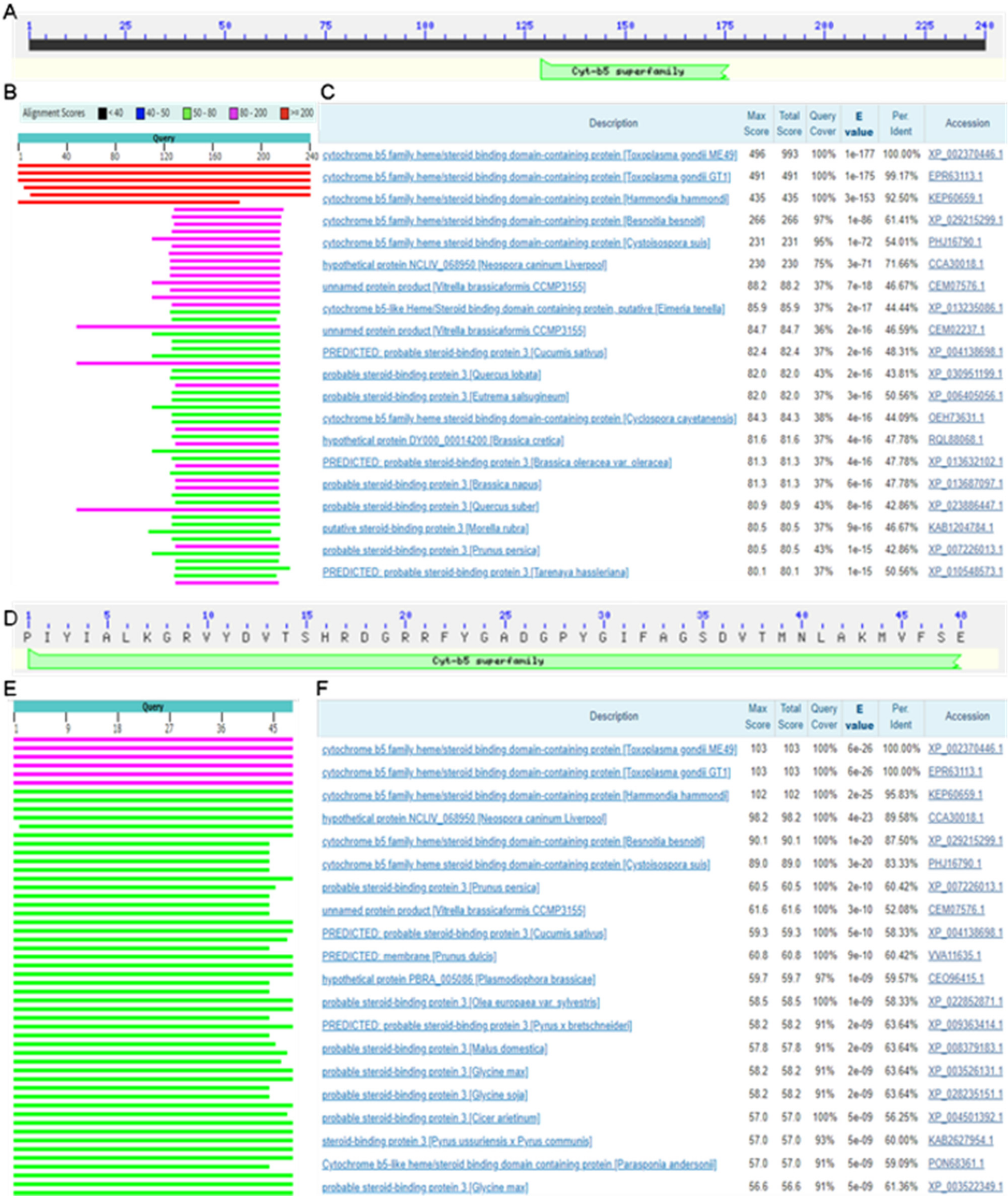

C

Description	Max Score	Total Score	Query Cover	E value	Per. Ident	Accession
cytochrome b5 family heme/steroid binding domain-containing protein [Toxoplasma gondii ME49]	496	993	100%	1e-177	100.00%	XP_002370446.1
cytochrome b5 family heme/steroid binding domain-containing protein [Toxoplasma gondii GT1]	491	491	100%	1e-175	99.17%	EPR63113.1
cytochrome b5 family heme/steroid binding domain-containing protein [Hammondia hammondi]	435	435	100%	3e-153	92.50%	KEP60659.1
cytochrome b5 family heme/steroid binding domain-containing protein [Besnoitia besnoiti]	266	266	97%	1e-86	61.41%	XP_029215299.1
cytochrome b5 family heme steroid binding domain-containing protein [Cystoisospora suis]	231	231	95%	1e-72	54.01%	PHJ16790.1
hypothetical protein NCLIV_068950 [Neospora caninum Liverpool]	230	230	75%	3e-71	71.66%	CCA30018.1
unnamed protein product [Vitrella brassicaformis CCMP3155]	88.2	88.2	37%	7e-18	46.67%	CEM07576.1
cytochrome b5-like Heme/Steroid binding domain containing protein, putative [Eimeria tenella]	85.9	85.9	37%	2e-17	44.44%	XP_013235086.1
unnamed protein product [Vitrella brassicaformis CCMP3155]	84.7	84.7	36%	2e-16	46.59%	CEM02237.1
PREDICTED: probable steroid-binding protein 3 [Cucumis sativus]	82.4	82.4	37%	2e-16	48.31%	XP_004138698.1
probable steroid-binding protein 3 [Quercus lobata]	82.0	82.0	43%	2e-16	43.81%	XP_030951199.1
probable steroid-binding protein 3 [Eutrema salsugineum]	82.0	82.0	37%	3e-16	50.56%	XP_006405056.1
cytochrome b5 family heme steroid binding domain-containing protein [Cyclospora cayetanensis]	84.3	84.3	38%	4e-16	44.09%	OEH73631.1
hypothetical protein DY000_00014200 [Brassica cretica]	81.6	81.6	37%	4e-16	47.78%	RQL88068.1
PREDICTED: probable steroid-binding protein 3 [Brassica oleracea var. oleracea]	81.3	81.3	37%	4e-16	47.78%	XP_013632102.1
probable steroid-binding protein 3 [Brassica napus]	81.3	81.3	37%	6e-16	47.78%	XP_013687097.1
probable steroid-binding protein 3 [Quercus suber]	80.9	80.9	43%	8e-16	42.86%	XP_023886447.1
putative steroid-binding protein 3 [Morella rubra]	80.5	80.5	37%	9e-16	46.67%	KAB1204784.1
probable steroid-binding protein 3 [Prunus persica]	80.5	80.5	43%	1e-15	42.86%	XP_007226013.1
PREDICTED: probable steroid-binding protein 3 [Tarenaya hassleriana]	80.1	80.1	37%	1e-15	50.56%	XP_010548573.1

F

Description	Max Score	Total Score	Query Cover	E value	Per. Ident	Accession
cytochrome b5 family heme/steroid binding domain-containing protein [Toxoplasma gondii ME49]	103	103	100%	6e-26	100.00%	XP_002370446.1
cytochrome b5 family heme/steroid binding domain-containing protein [Toxoplasma gondii GT1]	103	103	100%	6e-26	100.00%	EPR63113.1
cytochrome b5 family heme/steroid binding domain-containing protein [Hammondia hammondi]	102	102	100%	2e-25	95.83%	KEP60659.1
hypothetical protein NCLIV_068950 [Neospora caninum Liverpool]	98.2	98.2	100%	4e-23	89.58%	CCA30018.1
cytochrome b5 family heme/steroid binding domain-containing protein [Besnoitia besnoiti]	90.1	90.1	100%	1e-20	87.50%	XP_029215299.1
cytochrome b5 family heme steroid binding domain-containing protein [Cystoisospora suis]	89.0	89.0	100%	3e-20	83.33%	PHJ16790.1
probable steroid-binding protein 3 [Prunus persica]	60.5	60.5	100%	2e-10	60.42%	XP_007226013.1
unnamed protein product [Vitrella brassicaformis CCMP3155]	61.6	61.6	100%	3e-10	52.08%	CEM07576.1
PREDICTED: probable steroid-binding protein 3 [Cucumis sativus]	59.3	59.3	100%	5e-10	58.33%	XP_004138698.1
PREDICTED: membrane [Prunus dulcis]	60.8	60.8	100%	9e-10	60.42%	VVA11635.1
hypothetical protein PBRA_005086 [Plasmodiophora brassicae]	59.7	59.7	97%	1e-09	59.57%	CEO96415.1
probable steroid-binding protein 3 [Olea europaea var. sylvestris]	58.5	58.5	100%	1e-09	58.33%	XP_022852871.1
PREDICTED: probable steroid-binding protein 3 [Pyrus x bretschneideri]	58.2	58.2	91%	2e-09	63.64%	XP_009363414.1
probable steroid-binding protein 3 [Malus domestica]	57.8	57.8	91%	2e-09	63.64%	XP_008379183.1
probable steroid-binding protein 3 [Glycine max]	58.2	58.2	91%	2e-09	63.64%	XP_003526131.1
probable steroid-binding protein 3 [Glycine soja]	58.2	58.2	91%	2e-09	63.64%	XP_028235151.1
probable steroid-binding protein 3 [Cicer arietinum]	57.0	57.0	100%	5e-09	56.25%	XP_004501392.1
steroid-binding protein 3 [Pyrus ussuriensis x Pyrus communis]	57.0	57.0	93%	5e-09	60.00%	KAB2627954.1
Cytochrome b5-like heme/steroid binding domain containing protein [Parasponia andersonii]	57.0	57.0	91%	5e-09	59.09%	PON68361.1
probable steroid-binding protein 3 [Glycine max]	56.6	56.6	91%	5e-09	61.36%	XP_003522349.1

Figure S1. Alignment of the cytochrome b5 family heme/steroid binding domain-containing protein. A) Scheme of the primary sequence of cytochrome b5 family heme/steroid binding domain-containing protein, showing its Cyt b5 domain in green (aa 129–176). B) Classical protein Alignment. C) Data from the proteins that presented a significative alignment with the cytochrome b5 family heme/steroid binding domain-containing protein. D) Scheme of the primary sequence of cytochrome b5 family heme/steroid binding domain (aa 129–176). E) Classical protein Alignment of

the domain. F) Data from the proteins that presented a significative alignment with the cytochrome b5 family heme/steroid binding domain. Alignments were performed with the BLAST program from the NCBI website (https://blast.ncbi.nlm.nih.gov/Blast.cgi?PROGRAM=blastp&PAGE_TYPE=BlastSearch&LINK_LOC=blasthome)
